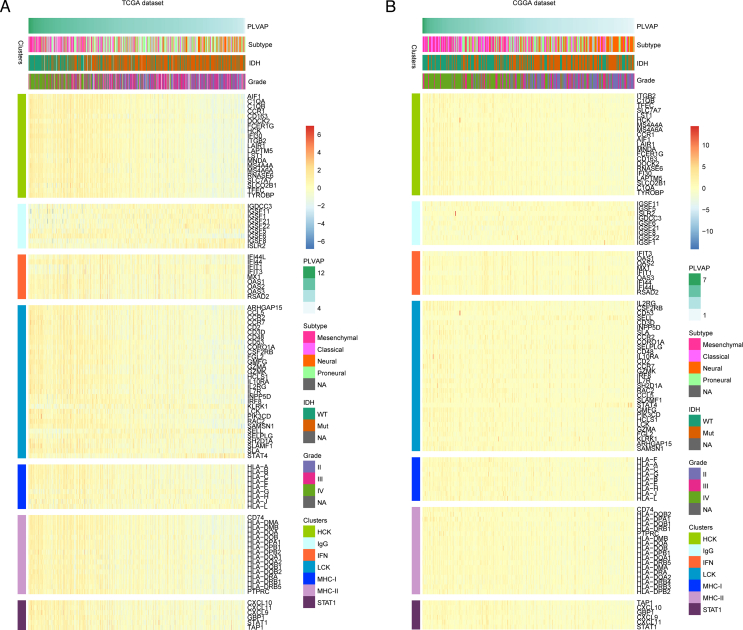# PLVAP is associated with glioma-associated malignant processes and immunosuppressive cell infiltration as a promising marker for prognosis

**DOI:** 10.1016/j.heliyon.2022.e10298

**Published:** 2022-08-19

**Authors:** Kaiming Ma, Xin Chen, Xiaofang Zhao, Suhua Chen, Jun Yang

**Affiliations:** a Department of Neurosurgery, Peking University Third Hospital, Beijing, China; b Center for Precision Neurosurgery and Oncology of Peking University Health Science Center, Beijing, China

**Keywords:** PLVAP, Biomarker, Gliomas, Target, Prognosis, Immunosuppression

## Abstract

Previous reports have confirmed the significance of plasmalemma vesicle-associated protein (PLVAP) in the progression of multiple tumors; however, there are few studies examining its immune properties in the context of gliomas. Here, we methodically investigated the pathophysiological characteristics and clinical manifestations of gliomas. A total of 699 patients diagnosed with gliomas in the cancer genome atlas along with 325 glioma patients in the Chinese glioma genome atlas were collected for the training and validation sets. We analyzed and visualized the total statistics using RStudio. PLVAP was markedly upregulated among high grade gliomas, O^6^-methylguanine-DNA methyltransferase promoter unmethylated subforms, isocitrate dehydrogenase wild forms, 1p19q non-codeletion subforms, and mesenchyme type gliomas. The receiver operating characteristics analysis illustrated the favorable applicability of PLVAP in regard to estimating mesenchyme subform gliomas. Subsequent Kaplan–Meier curves together with multivariable Cox analyses upon survival identified high-expression PLVAP as a distinct prognostic variable for patients with gliomas. Gene ontology analysis of PLVAP among gliomas has documented the predominant role of this protein in glioma-associated immunobiological processes and also in inflammatory responses. We consequently examined the associations of PLVAP with immune-related meta-genes, and PLVAP was positively correlated with hematopoietic cell kinase, lymphocyte-specific protein tyrosine kinase, major histocompatibility complex (MHC) I, MHC II, signal transducer and activator of transcription 1, and interferon and was negatively correlated with immunoglobulin G. Moreover, association analyses between PLVAP and glioma-infiltrating immunocytes indicated that the infiltrating degrees of most immune cells exhibited positive correlations with PLVAP expression, particularly immunosuppressive subsets such as tumor-related macrophages, myeloid-derived suppressor cells, and regulatory T lymphocytes. In summary, we originally demonstrated that PLVAP is markedly associated with immunosuppressive immune cell infiltration degrees, unfavorable survival, and adverse pathology types among gliomas, thus identifying PLVAP as a practicable marker and a promising target for glioma-based precise diagnosis and therapeutic strategies.

## 1 Introduction

Gliomas are intractable idiopathic neoplasms that comprise the largest proportion of the central nervous system (CNS) [1, 2]. The latest research reported that the incidence of gliomas reached 80.8% of the overall incidence of CNS essential malignancy, while the death toll from gliomas was 88.1% of the total fatalities due to CNS neoplasms [2]. For such complex neoplasms, the currently approved clinical therapeutic protocols include surgery remedies, chemotherapeutic drugs, radiotherapeutics, and also several burgeoning adjunctive therapeutics such as molecule-targeted therapeutics, immunological approaches, and tumor treating fields (TTFs) [3, 4]. Nonetheless, these therapeutics alone or in combination are still unsatisfactory after verification with clinical practical application, notably ameliorating the overall prognosis for patients diagnosed with gliomas, particularly for high-grade gliomas (HGG) [4, 5, 6]. The most recent version of the World Health Organization (WHO) classification of CNS tumors reaffirmed that molecular biomarkers are essential for providing practical evidence for glioma-based diagnosis and healing [1]. Therapeutic strategies that integrate more precise glioma-related biomarkers possess the potential to surmount existing therapeutic woes [5]. Hence, identifying more precise biomarkers that are inextricably linked to glioma clinicopathology and progression is imperative [7].

**Fig. S1:**
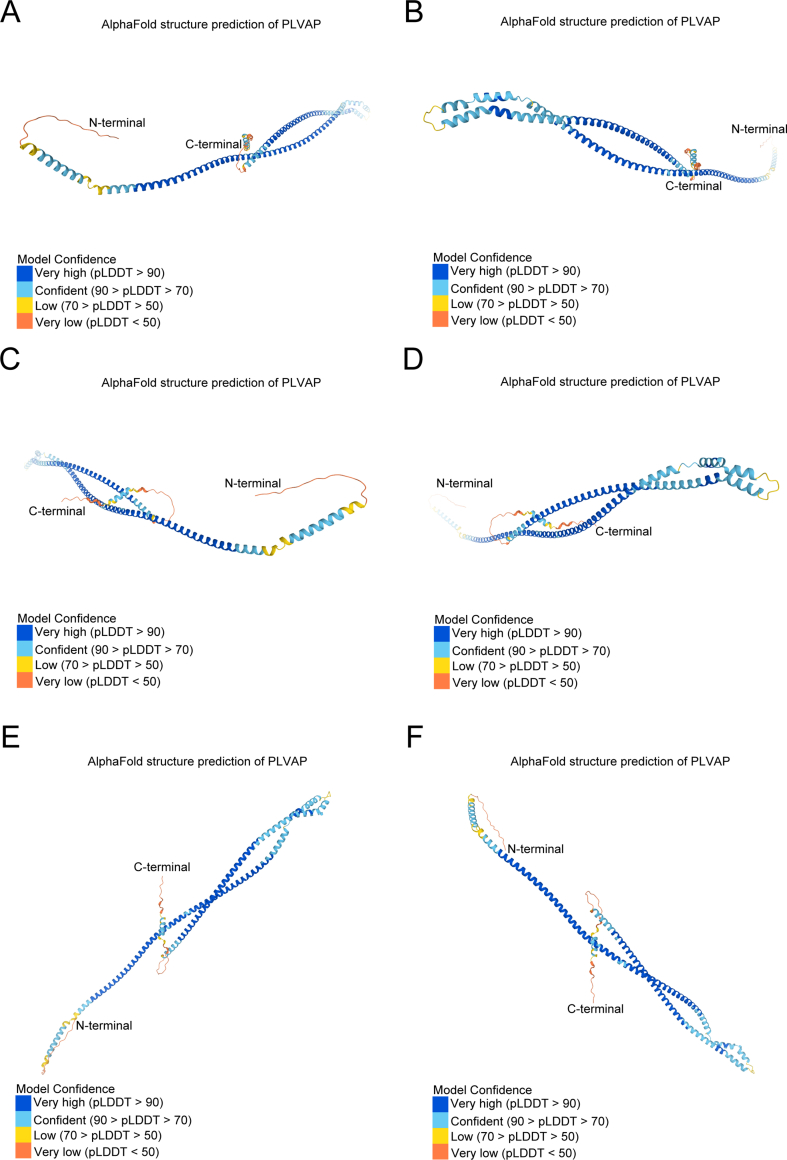


Plasmalemma vesicle-associated protein (PLVAP), corresponding to PV-1, FELS, and MECA32, acts as an endotheliocyte type II transmembrane glycoprotein with a molecular weight of 55–65 kDa that *in situ* forms rod-like heparin-binding homodimeric fibrils [8, 9]. The extracellular domain is composed of PLVAP bulk [9], and glycosylation near the transmembrane domain can prevent PLVAP dimers from collapsing, while the intracellular domain connects with the cytoskeleton through direct binding or cytoskeletal linking molecules to stabilize PLVAP on the membrane [9, 10]. We put forward the three-dimensional structure of human PLVAP using AlphaFold [11](Figure S1), and the structure consists of a simple endocellular tail and one single membrane spanning area together with one complex exocytic domain, while the amino-terminal and carboxyl-terminal regions are primarily composed of low-complexity unstructured regions (per-residue confidence score [pLDDT] < 50). This structural analysis is similar to previous results [9, 12, 13]. As the only known molecule forming diaphragms for fenestra and stomata, PLVAP predominantly constitutes the caveolar∗∗ diaphragm [9, 14] that is required for fenestral pore structure and the orderly organization of fenestra in sieve plates [15]. PLVAP is primarily enriched in the capillaries and venules of the lungs, kidneys, liver, and certain tumors [16, 17]. In the normal brain, PLVAP is limited among the vasculatures of the plexus chorioideus together with the circumventricular organ where endotheliocytes of the blood brain barrier (BBB) are fenestrated to enable interchange between the cerebrospinal fluid and blood [18]. The major physiological function of PLVAP is to regulate the permeability and homeostasis of blood vessels, transendothelial transport, and the promotion of angiogenesis [13, 17, 19]. PLVAP is also correlated with caveolae-mediated transcytosis and the transportation of intracellular albumin to the extracellular matrix or interstitial spaces [14].

Regarding pathological processes, PLVAP actively participates in damage to the vascular barrier [20], vasogenic edema [14], proinflammatory response [13, 14, 21, 22], chronic hypoxia [23], tumorigenesis [14], and pathological degradation of paracellular tight and adherens junctions in the BBB [9, 20]. PLVAP is significantly upregulated in chylous diarrhea-related hepatic damage [24], pulmonary fibrosis [25], acute stroke [26], traumatic spinal cord injury [27], schizophrenia [20], and a number of cancers. Recent reports have verified PLVAP as a practical marker in addition to a prospective treatment targeting lung cancer [28], cholangiocarcinoma [19], colorectal cancer (CRC) [29], pancreatic adenocarcinoma [30], melanomas [31], liver cancer [32] and brain tumors [33, 34]. Recent studies have confirmed that glioma cells specifically stimulate the expression of PLVAP [26]. PLVAP is overexpressed in glioblastoma (GBM) both at the mRNA [34] and protein levels [33, 35] but is rarely expressed in normal brain tissues, and this has also been verified in U87 mouse xenograft models [36]. It was also demonstrated that PLVAP is consistently distributed from the core to the edge of the GBM [26]. As a glioma endothelial marker gene [34], PLVAP was deemed to act as a novel biomarker of angiogenesis and BBB integrity in gliomas and was also identified as a possible therapeutic target [32, 33, 37]. However, previous studies have primarily focused on GBM, and no large-scale clinical analysis of cases has revealed the comprehensive characteristics of PLVAP in the context of gliomas. Moreover, there are few studies examining the immune properties of PLVAP in gliomas, particularly in the context of immunosuppressive processes, thus making it difficult for us to objectively recognize its role and deeply restricting the clinical translation of PLVAP-targeted therapies for glioma.

Given the above limitations, here we methodically investigated PLVAP in the context of glioma from its expression patterns, pathobiological roles, and prognosis significance with the most large-scale clinical cases. Notably, we placed emphasis on the characteristics of PLVAP in glioma-relevant inflammatory and immunosuppressive responses. A total of 699 patients diagnosed with gliomas from the Cancer Genome Atlas (TCGA) along with 325 glioma patients from the Chinese glioma genome atlas (CGGA) were correspondingly collected for the training set and validation set based on ribonucleic acid sequencing (RNA-seq) data. Our exploration expands the possibility of PLVAP-targeted tactics for glioma-based precise diagnosis and therapies.

## 2 Results

### 2.1 PLVAP is indicated to be markedly upregulated among high grade, isocitrate dehydrogenase (IDH) wild form, non-codeleted 1p19q subform in combination with unmethylated O^6^-methylguanine- deoxyribonucleic acid methyltransferases (MGMT) promoter subform gliomas

**Figure 1:**
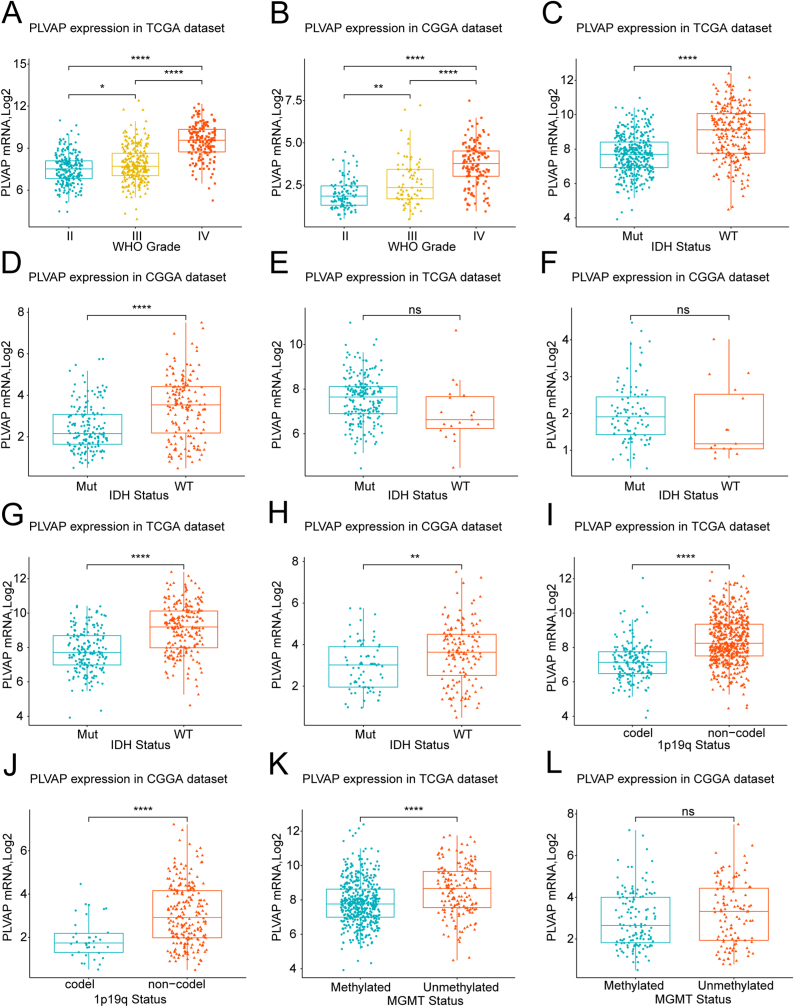
PLVAP expression patterns for gliomas among various WHO grades (A, B), isocitrate dehydrogenase forms (all grades [C, D], low grades [E, F], high grades [G, H]), and 1p/19q-codeletion statuses (I, J) and O^6^-methylguanine-methylguanine-deoxyribonucleic acid methyltransferases promoters statuses (K, L). PLVAP is markedly upregulated among high grades, isocitrate dehydrogenase wild form, and non-codeleted 1p19q subform along with unmethylated O^6^-methylguanine-deoxyribonucleic acid methyltransferase promoters subform gliomas. ∗P value below 0.05, ∗∗P value below 0.01, ∗∗∗P value below 0.001, ∗∗∗∗P value below 0.0001.

First, we studied the corresponding RNA-seq information from these glioma cases in both sets to investigate the PLVAP-expression status. PLVAP was remarkably overexpressed in gliomas classified as high WHO grades, particularly among patients with GBM in both cohorts (Figure 1A and B). Moreover, the status of IDH mutation, 1p19q-codeletion, and MGMT methylation have already been verified to be essential in regard to defining glioma types and predicting the survival of patients [1]. Thus, we compared the variance in the mRNA levels of PLVAP among the molecular pathological types of gliomas. Our analysis results revealed notably higher levels of PLVAP expression among IDH wild forms, non-codeleted 1p19q subforms, and unmethylated MGMT promoter subforms in comparison to levels in gliomas of the IDH mutation subtype (Figure 1C and D for total WHO grades; E, F for low WHO grade; G, H for high WHO grades) and the co-deleted 1p19q subform (Figure 1I and J) in combination with methylated MGMT promoter subtype (Figure 1K and L) according to the TCGA and CGGA databases. Therefore, the above results revealed that PLVAP is overexpressed among these poor molecular pathological subtypes, and this expression status acts as an adverse biomarker for therapy reactivity and overall prognosis of glioma.

### 2.2 High expression of PLVAP relates to a worse prognosis for patients with glioma

**Figure 2:**
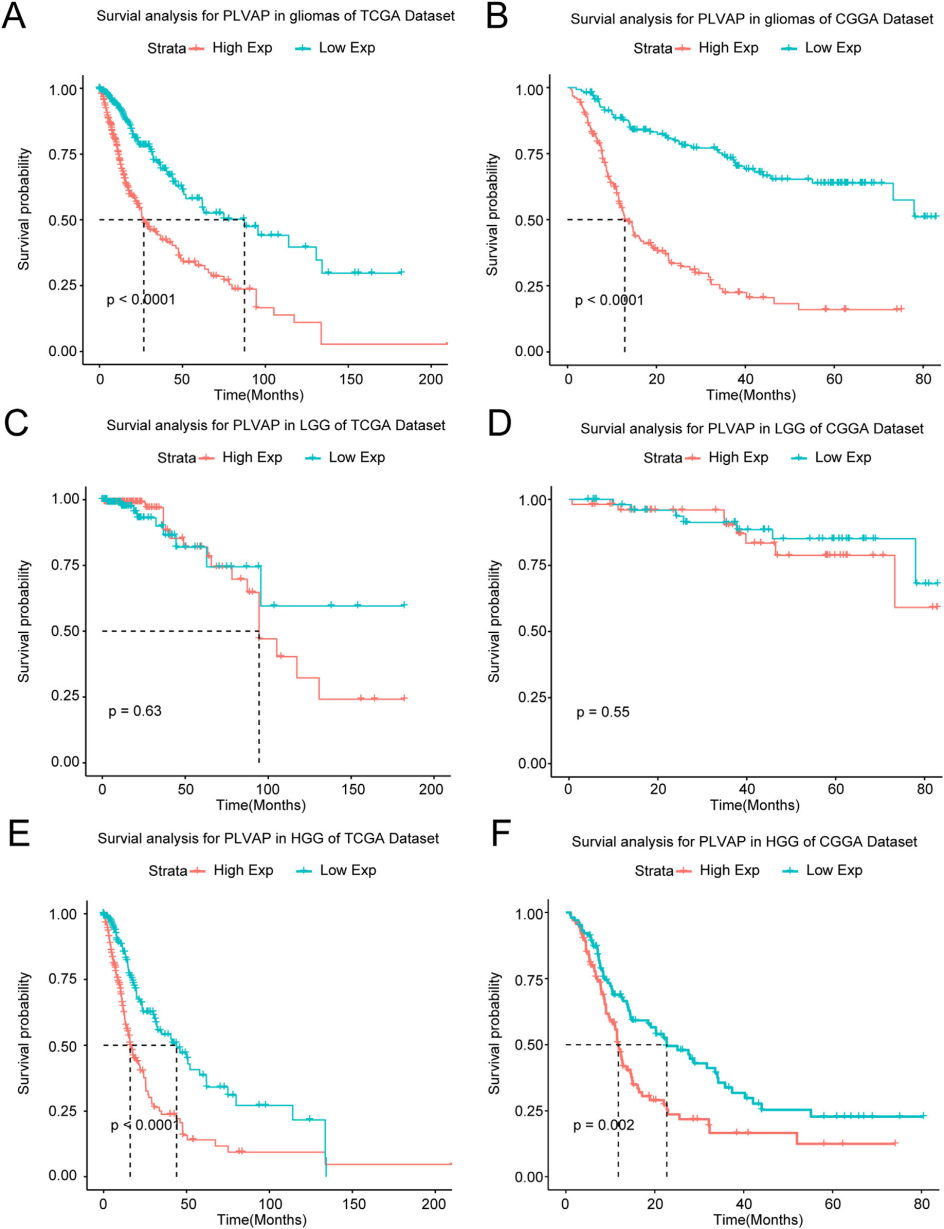
Kaplan–Meier survival curvilinear analysis for PLVAP among patients with overall grades (A, B), low grade (C, D), and high grade (E, F) gliomas. Highly-expressed PLVAP is related to worse prognosis for patients with glioma, particularly for patients with high grades gliomas.

**Figure 3:**
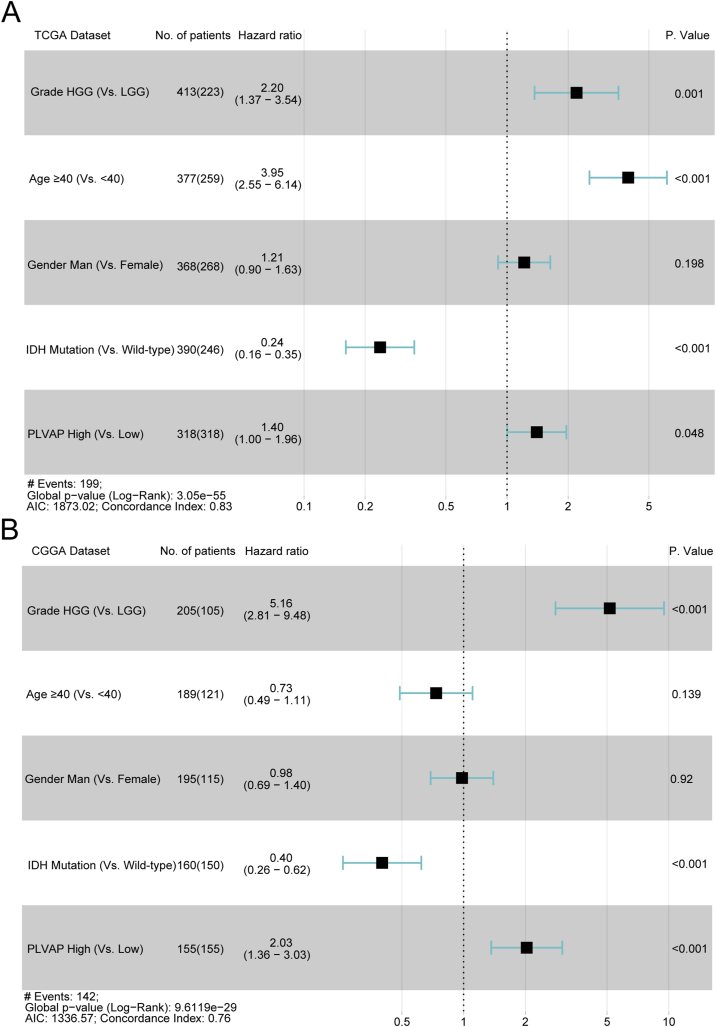
Multivariable Cox analyses of PLVAP among glioma patients (A, B). High expression of PLVAP is a distinct prognostic variable for patients with gliomas compared to gender, age, WHO grades, and isocitrate dehydrogenase statuses.

These findings demonstrated that PLVAP may provide a possible biomarker for malignant gliomas. Subsequently, we explored the relationship between PLVAP and the prognosis of glioma patients. First, we plotted the Kaplan-Meier models using the survival statistics from glioma samples from the TCGA and CGGA sets (Figure 2A and B), and those patients with highly expressed PLVAP exhibited significantly shorter overall survival (OS) time (P < 0.0001). Second, we separately analyzed the impact of PLVAP on the OS rate for patients with low-grade gliomas (LGG) (Figure 2C and D) as well as patients with HGG to avoid the effects of heterogeneous differences in various tumoral grades, and we determined that the impact of PLVAP on OS was more apparent for HGG patients (Figure 2E and F). Moreover, we further investigated the role of PLVAP in the overall prognosis of glioma patients interconnected with certain major clinical variables such as sex, age, WHO grades, and IDH mutant status (Figure 3A and B). The above survival analyses using multivariate COX statistics further confirmed that high expression of PLVAP is a risk factor and also an adverse marker that is correlated with worse survival in glioma patients.

### 2.3 PLVAP is remarkably upregulated in mesenchymal subtype glioma and is applicable for estimating the mesenchyme subform

**Figure 4:**
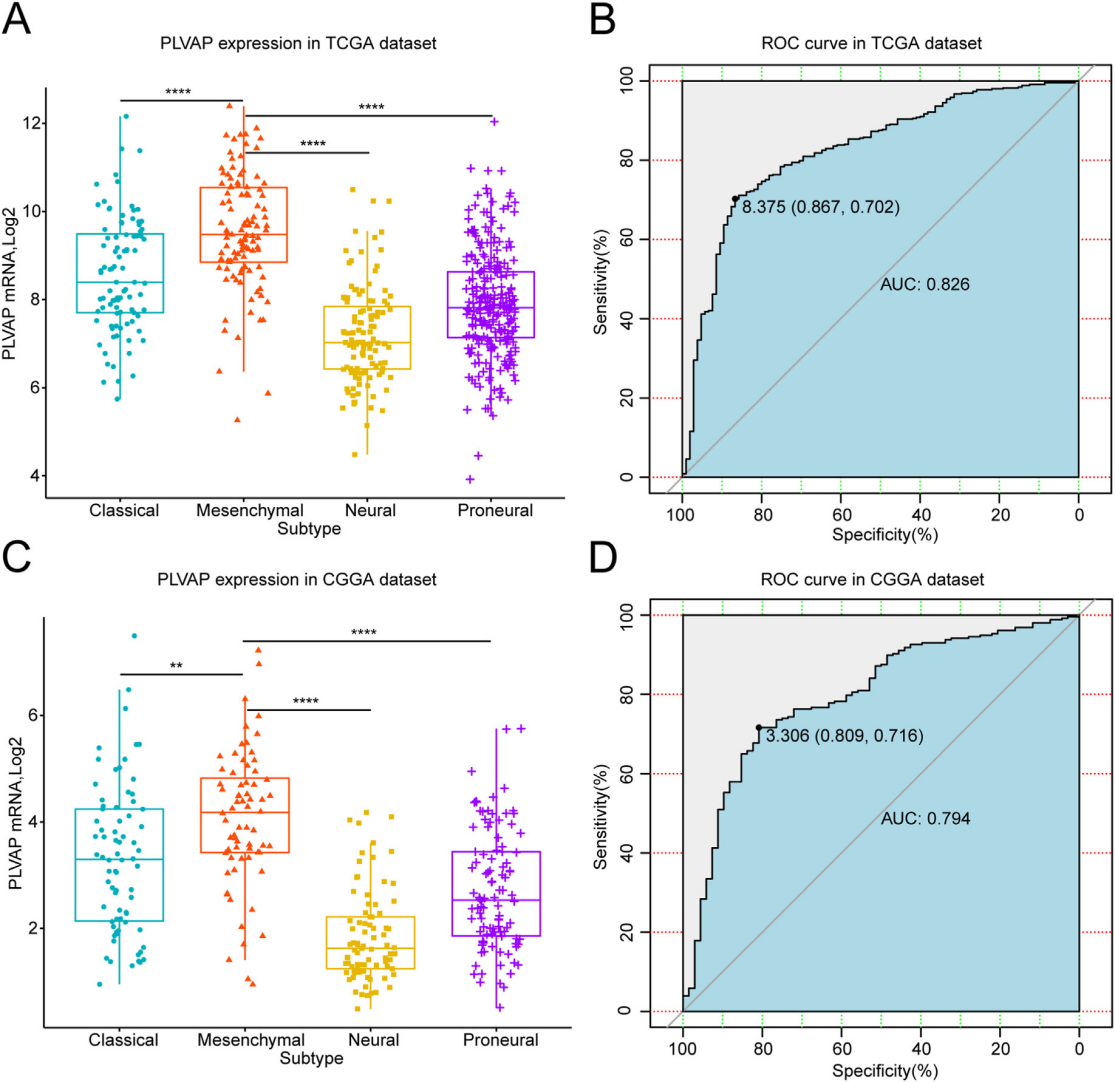
Relation between PLVAP expression and TCGA subtypes of glioma. PLVAP is indicated to be markedly upregulated in the mesenchyme subtype of both sets (A, C). Receiver operation characteristics analysis revealed the favorable applicability and accuracy of PLVAP in estimating mesenchyme subform gliomas (B, D). ∗P value below 0.05, ∗∗P value below 0.01, ∗∗∗P value below 0.001, ∗∗∗∗P value below 0.0001.

Throughout the years, the TCGA system has classified gliomas as mesenchymal subforms along with three other molecule-based subforms [38]. This categorization was verified as consequential to patient survival duration, particularly the mesenchyme subform that represented adverse progression together with worse survival of tumors [6]. Subsequently, we studied the relationship between PLVAP expression and the four subtypes in the two datasets (Figure 4). PLVAP was markedly upregulated in the mesenchymal subtype in both sets (Figure 4A and C). Next, receiver operating characteristics (ROC) analysis was utilized to estimate the favorable applicability of PLVAP in estimating mesenchyme subform gliomas. For the TCGA database (Figure 4B), the area under the curve reached 0.826 when PLVAP predicted gliomas of the mesenchyme subform. Meanwhile, the corresponding specificity was 86.7 percent, and the sensitivity was 70.2 percent with an optimal cut-off value of 8.375. Similarly, the area under the curve reached 0.794 for the CGGA database (Figure 4D), and the corresponding specificity and sensitivity were 80.9% and 71.6 %, respectively, with an optimal cutoff value of 3.306. The above statistics revealed the favorable applicability and accuracy of PLVAP in estimating mesenchyme subform gliomas.

### 2.4 PLVAP is linked to glioma-associated immunizing responses

**Figure 5:**
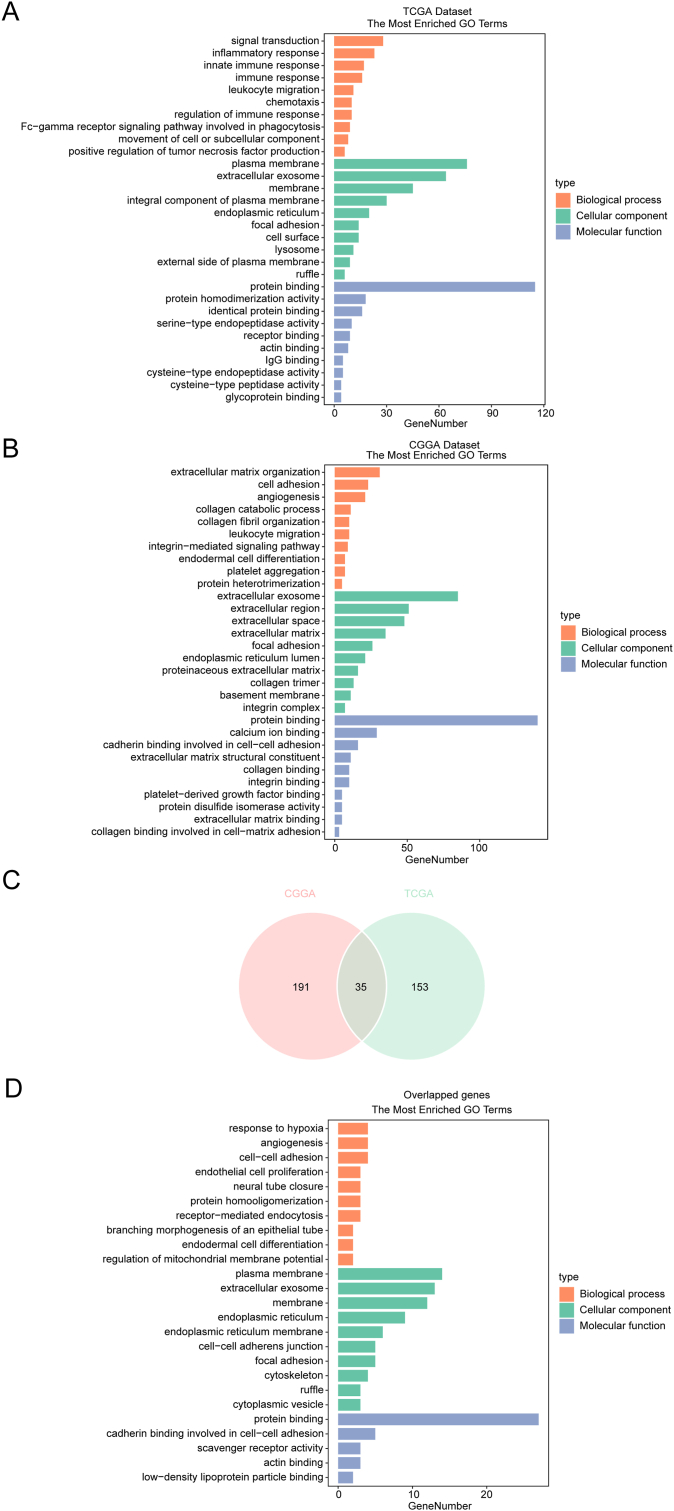
Gene ontology analysis of PLVAP in gliomas among the TCGA set (A), the CGGA set (B), and the 35 overlapped genes of both sets (C, D). PLVAP is closely involved in glioma-associated immune activities and inflammation responses, predominantly in the extracellular space or on cell membranes as extracellular exosomes, membrane components, and integrin complex components.

For the purpose of deeply exploring these characteristics along with the biological functions of PLVAP, we sequenced PLVAP-related genes according to Spearman correlation analysis (Supplementary Table 1). For the TCGA dataset, we finally filtered out 188 relevant genes for those with absolute correlation coefficient values of greater than 0.6 (P < 0.05), and among these, 170 exhibited a positive correlation with PLVAP, while 18 exhibited a negative correlation. Using the same criterion, 226 relevant genes were filtered out for the CGGA dataset, and among these, 206 exhibited a positive correlation with PLVAP, while 20 exhibited an opposite correlation. Subsequent gene ontology analyzation of these genes was conducted using the DAVID website (Figure 5A and B). PLVAP-involved biological procedures primarily include immune responses, inflammatory responses, inherent immunity responses, immune response regulation, leukocyte migration and cell adhesion, chemotaxis, signal transducting and integrin-mediated signaling pathway, angiogenesis, endodermal cell differentiation and proliferation, extracellular matrix (ECM) organization and collagen catabolic process, and response to hypoxia. Regarding cellular components, PLVAP primarily acts as the constituent part of cellular membranes, adhesion plaques, and integrin complexes that are located on the cell envelope, plasma membranes, endoplasmic reticulum, lysosomes, and extracellular space (ECS). Several major molecular functions of PLVAP include protein and receptor binding, calcium ion and integrin binding, cysteine-type endopeptidase and peptidase activity, ECM binding, and collagen combined with cell-matrix adhesion. These findings were consistent for both the TCGA and CGGA cohorts. Furthermore, gene ontology analysis of these 35 correlated genes from both datasets was performed to provide an additional validation as presented in Figure 5C and D and in Supplementary Table 2. This work demonstrated the involvement of PLVAP in glioma-associated immune and inflammatory responses, predominantly in the ECS or on cell membranes as extracellular exosomes, membrane components, and integrin complexes.

**Figure 6:**
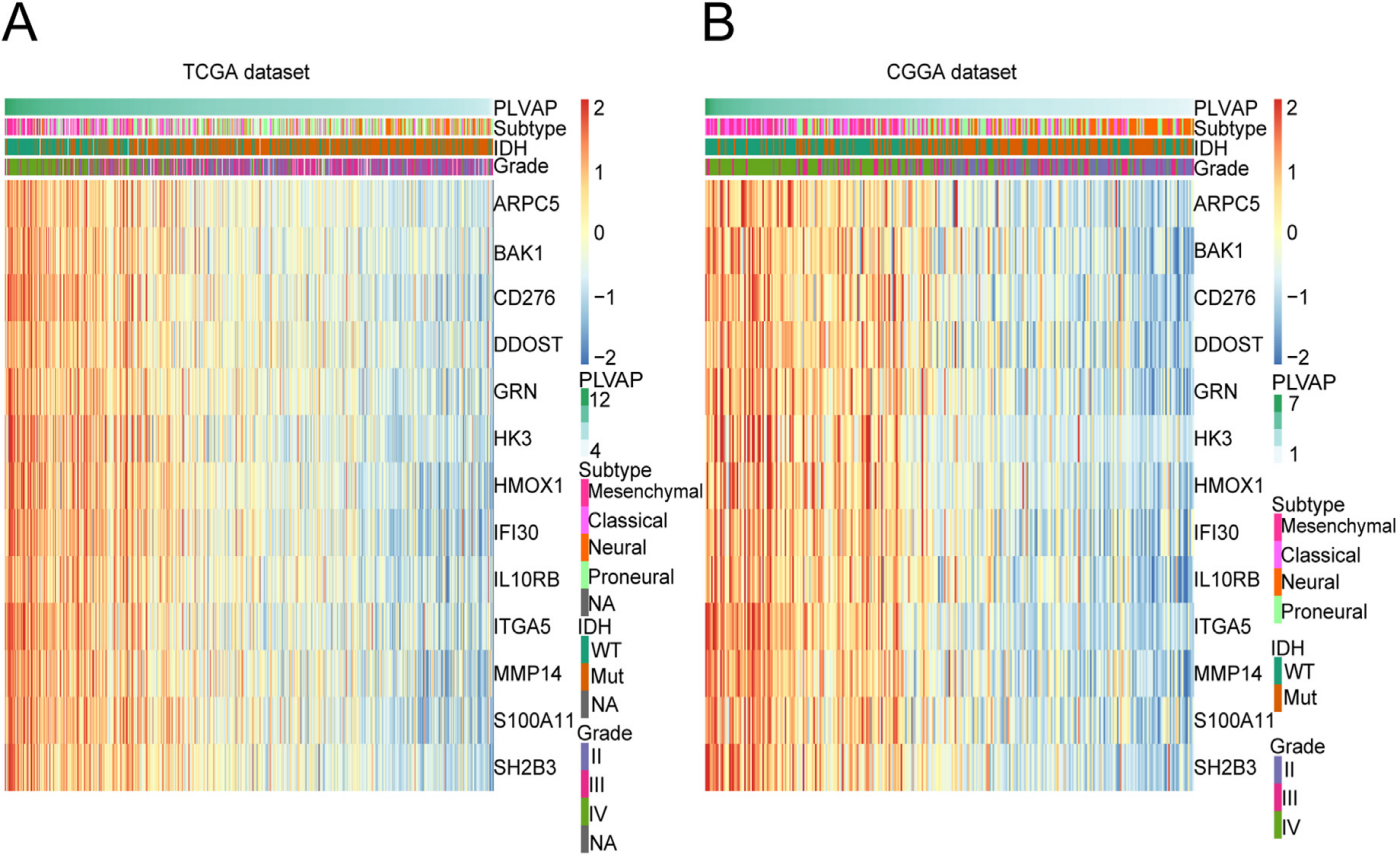
Heatmap analysis of correlations among immunogene subsets and PLVAP for glioma (A, B). Immunizing genes predominantly exhibit a positive correlation with PLVAP for both datasets, thus revealing the role of PLVAP in the context of glioma-associated immune responses.

To investigate the functions of PLVAP in the context of glioma-associated immune responses, these immunogene subsets were downloaded from the AmiGO2 website. Based on these subsets, 100 genes in the TCGA dataset and also 60 genes from the CGGA set that were notably related to PLVAP (|R| > 0.6, P < 0.05) were selected to perform heatmap analysis (Supplementary Table 3). Then the overlapped 13 immunizing genes of them were used to draw the heat map (Figure 6A and B). Finally, we concluded that the above immunizing genes exhibit a positive correlation with PLVAP in both datasets, thus further revealing the role of PLVAP in glioma-associated immune responses.

### 2.5 PLVAP is strongly relevant to the inflammation activities of gliomas

**Figure 7:**
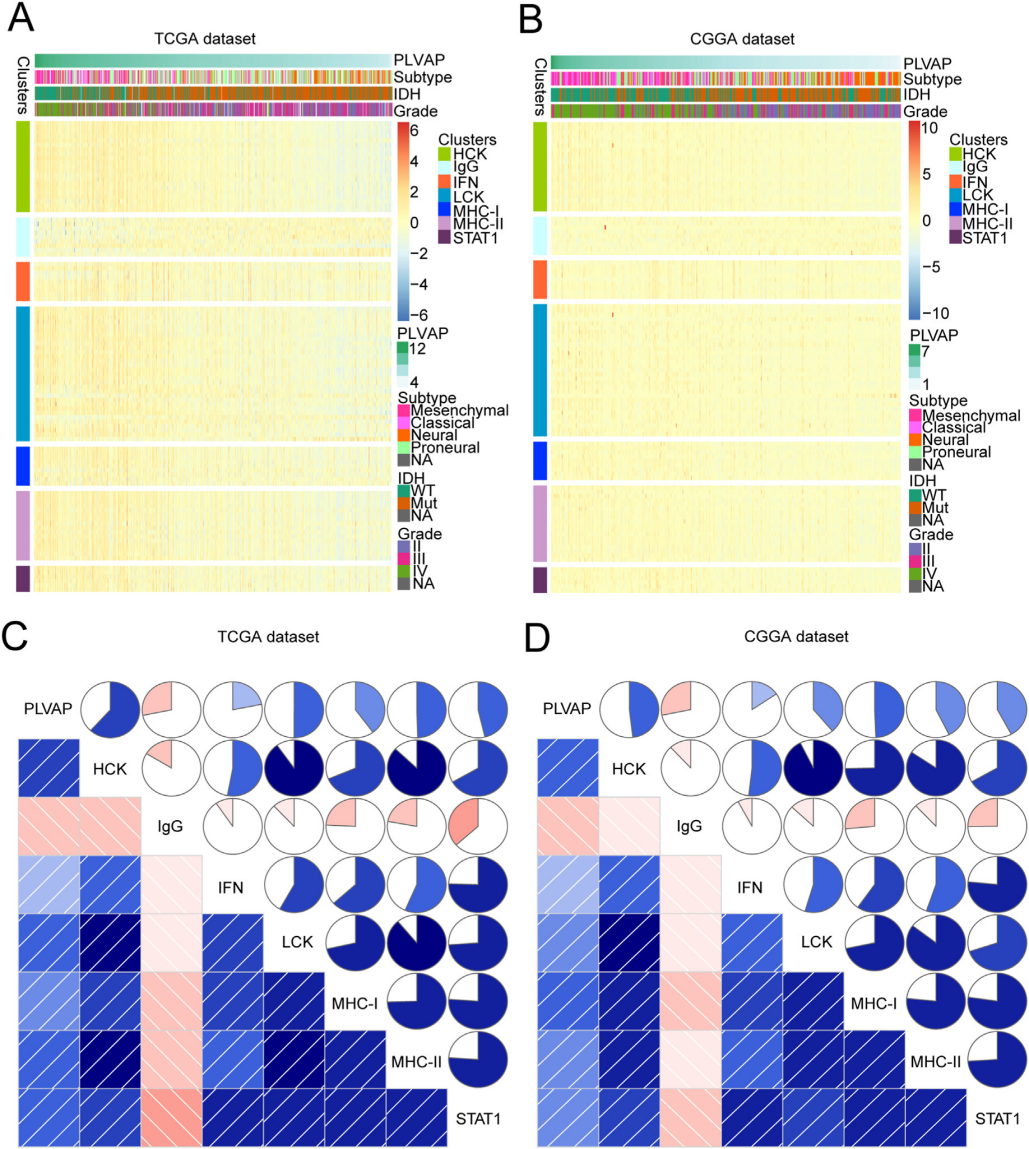
Inflammation-related functions of PLVAP among gliomas. Heatmap analysis of correlations of inflammatory genes with PLVAP expression (A, B). Genes set variant analysis of PLVAP and inflammatory metagenes (C, D). Blue indicates positive correlation, while red indicates negative correlation. Bicolor gradation together with the circle dimension are in proportion to correlation degree. PLVAP expression is significantly positively correlated with hematopoietic cell kinase (HCK), lymphocyte-specific protein tyrosine kinase (LCK), major histocompatibility complex (MHC) I, MHC II, signal transducer and activator of transcription 1 (STAT1), and interferon (IFN), while immunoglobulin G (IgG) is negatively correlated.

As stated based on the above experiments, PLVAP is associated with inflammatory responses in glioma. Therefore, to search for specific inflammation-related functions of PLVAP, we included 104 inflammation genes that could generally fall into seven metagenes [6]. Supplementary Table 4 contains the specified lists of these metagenes. In the CGGA and TCGA databases, heatmap analysis (Figure 7A and B; Figure S2A, B) of those inflammatory metagenes demonstrated their correlations with PLVAP, and immunoglobulin G (IgG) metagenes exhibit an inverse connection with PLVAP, while the other six metagenes exhibited an opposite connection. For further confirmation, gene set variant analysis of PLVAP and also the metagenes described above was conducted to plot corresponding correlograms based on Pearson correlation analysis (Figure 7C and D). The above analyses among the TCGA and CGGA databases were highly coherent with the heatmaps. We verified that PLVAP expression is significantly positively correlated with hematopoietic cell kinase (HCK), lymphocyte-specific protein tyrosine kinase (LCK), major histocompatibility complex (MHC) I, MHC II, signal transducer and activator of transcription 1 (STAT1), and also with interferon (IFN); however, the results for IgG were the opposite.

### 2.6 Association analyses examining PLVAP in relation to glioma-infiltrating immunocytes

**Figure 8:**
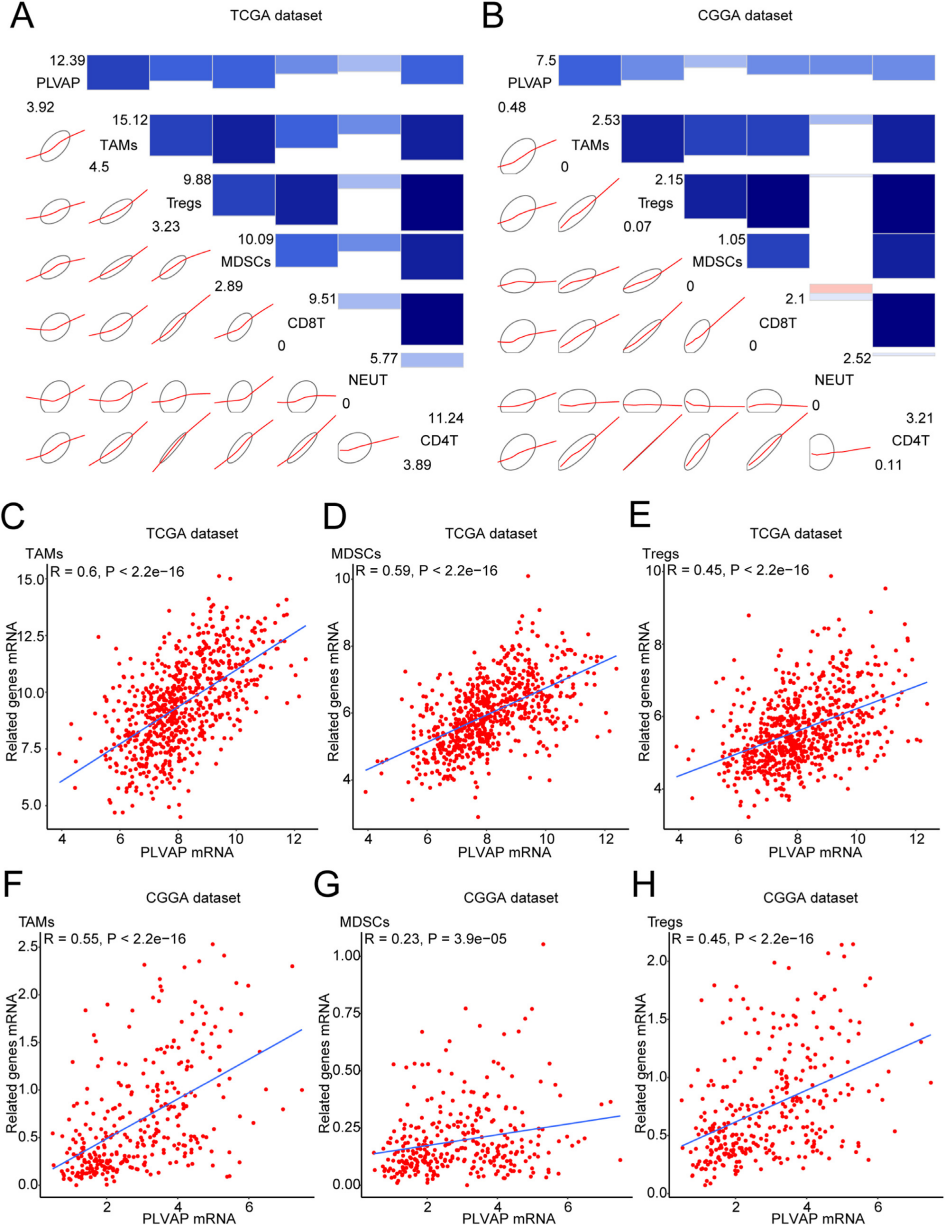
Associations analyses of PLVAP and gliomas-infiltrating immunocytes. Corrgram analyses visualizing associations of PLVAP with six major tumor-infiltrating immunocyte subpopulations (A, B). Blue indicates positive correlation, while red indicates negative correlation. Bicolor gradation together with the circle dimension are in proportion to correlation degree. The leading diagonal contains the minimum and maximum values of the variables. Spearman associations analyses of PLVAP and tumor-associated macrophages (TAMs) (C, F), myeloid-derived suppressor cells (MDSCs) (D, G) and regulatory T lymphocytes (Tregs) (E, H) among the two datasets. Cases of glioma are displayed as dots, and regression analyzing lines are added into corresponding points in the diagram.

The infiltration of immunocytes into tumors has been verified as a key component of the immunosuppressive microenvironment and also the invasive processes of malignant gliomas [39]. Thus, further analyses examining the association between PLVAP and glioma-infiltrating immunocytes are indispensable. We selected six major tumor-infiltrating immunocyte subpopulations for analysis (Supplementary Table 5). Later, corrgram analyses were performed to visualize associations of PLVAP with the above immunocyte subpopulations among the TCGA and CGGA datasets (Figure 8A and B), and our results indicated that the infiltration degrees for most immunocytes were positively correlated with PLVAP expression. Moreover, Spearman association analyses indicated that PLVAP was positively correlated with immunosuppressive subsets such as tumor-associated macrophages (TAMs) (Figure 8C and F), myeloid-derived suppressor cells (MDSCs) (Figure 8D and G), and regulatory T lymphocytes (Tregs) (Figure 8E and H) among those two datasets. For TAMs, the correlation coefficient values for the TCGA and CGGA sets were 0.6 and 0.55. For MDSCs, the correlation coefficient values for the TCGA and CGGA sets were 0.59 and 0.23. For Tregs, the correlation coefficient values for the TCGA and CGGA sets were 0.45 and 0.45.

## 3 Discussion

Although a number of clinical trials examining glioma therapies have not led to any satisfying advances based on targeted therapies, novel therapeutic strategies integrating adequate practical biomarkers inextricably linked to glioma clinicopathology are encouraging in regard to surmounting existing treatment woes [5, 6]. This study confirms that overexpressed PLVAP is correlated with some adverse pathology types and angiogenesis process of glioma. More importantly, compared with previous studies, we originally revealed that PLVAP is markedly associated with immunosuppressive immunocyte infiltration degrees, inflammatory responses, and unfavorable survival of gliomas, which suggests that PLVAP may serve as a practicable marker and a promising target for glioma-based immunotherapy combined with anti-angiogenesis therapy.

Firstly, our results indicated that PLVAP is related with IDH wild-type, non-codeleted 1p19q, unmethylated MGMT promoter, and mesenchyme subforms of gliomas, which was little known in previous researches. Additionally, we identified high-expression PLVAP as a distinct prognostic variable for gliomas, while earlier reports had displayed similar findings in the context of CRC [29] and cholangiocarcinoma [19]. Based on the recognized clinical significance of these molecular pathological subforms for glioma, glioma patients with elevated PLVAP expression in tumor tissue may possess a high probability of neoplasm invasion, local recurrence, and treatment insensitivity. Earlier reports demonstrated that PLVAP in the endotheliocytes of GBM was selectively and stably expressed compared to expression in peritumoral brain tissue [33, 35]. The expression levels of PLVAP were markedly induced among endotheliocytes when exposed to culture media from the GBM cell lines U87MG and U251MG [33]. Several biological factors, signaling pathways, and physiological stimuli have been identified as key regulators of PLVAP, including VEGF [17, 40, 41], endothelial protein caveolin-1 ^13^, angiotensin II [42], transforming growth factor β [43], tumor necrosis factor α [43], phorbol myristate acetate [44], Wnt-beta-catenin signaling [45, 46], Notch signal pathways [47], fibronectin-integrin signaling [48], shearing force [43] and high glucose environment [49]. The increase in PLVAP in gliomas was not only due to the increase in endotheliocytes but also to the induction of tumor-related genes [26] that was primarily mediated through Wnt and the Notch signaling pathway [50]. PLVAP is actively involved in remodeling and leakage of the BBB in gliomas [26]. The expression of PLVAP induced by tumor cells increases the permeability of vessels by accelerating transcytosis of endotheliocytes through the founding trans-endothelial channel, fenestra, and diaphragm of the caveola [18]. Beyond the revulsant of diaphragm archebiosis, PLVAP also adjusts the quantity of fenestra and caveola through a VEGF-dependent approach and promotes transendothelial transport in endotheliocytes [9, 51, 52]. PLVAP-induced disruption of the BBB causes massive leakage of water and plasma proteins from cerebral vessels into the ECS [53, 54], and this results in ECS loosening and peritumoral brain edema (PTBE) from gliomas [9, 33]. Meanwhile, PLVAP promoted tumoral neovascularization with high permeability through its interaction with VEGF [17], and PLVAP knockdown remarkably reduced angiogenesis and tumor growth of gliomas *in vivo* and in *vitro* [33]. PLVAP is also involved in functional disorder and angiogenic remodeling of the BBB caused by chronic hypoxia in gliomas [23]. PLVAP-mediated BBB remodeling, expanding the ECS of PTBE, and neovascularization in gliomas provided ideal nutritional support for tumor cells and formed an invasive niche suitable for the progression of gliomas [55]. These studies also further support our findings that PLVAP could be regarded as a practical marker for glioma-based evaluation of molecular pathological subforms and also of long-term survival.

Secondly, we illustrated the predominant role of PLVAP among glioma-relevant immunobiological processes, inflammatory responses, angiogenesis process, and also ECM organization. Moreover, we uncovered that PLVAP is correlated with the infiltration of TAM, MDSC, and Tregs, which had not been reported before. These inspiring explorations reveal the potential involvement of PLVAP in glioma-connected immunosuppression along with invasion. Previous studies confirmed that PLVAP could aggravate inflammatory response by facilitating leukocyte trafficking via transcellular paths [13, 21, 22]. Leukocytes can extravasate through PLVAP-induced transendothelial channels in response to inflammation [22]. The interaction between PLVAP and vimentin promotes leukocyte trafficking [22]. PLVAP blockade was demonstrated to impair the recruitment of NEUT and macrophages during inflammatory activities [22]. PLVAP-blocking with MECA-32 antibody *in vivo* greatly reduced leukocyte migration by 85% in acute peritonitis models [22]. Furthermore, *in vitro* transmigration experiments have demonstrated that PLVAP redistributes in the presence of vimentin and caveolin-1 and partially colocalizes with the surrounding migrating lymphocytes [22]. PLVAP could offer the path of least resistance for lymphocytes and thus control the migration and selective entrance into the lymphaden [56]. The PLVAP-formed diaphragm in the transendothelial channel creates physical sieves that regulate the dimension-limited entrance for lymphatic-carried microsomes inside lymph reticulation systems [56]. Anti-PLVAP antibodies significantly inhibited the transmigration of lymphocytes through the endotheliocyte layer, while the rolling and adhesion processes were not affected [22]. PLVAP in endotheliocytes is essential for the renewal and maintenance of IgM+ B lymphocytes in enterocoelia and the spleen [21]. PLVAP is actively related to the recruitment of neoplasm-infiltrated leukocytes and also to neoplasm growth promoting of pancreatic adenocarcinoma xenografts [30]. Thus, the involvement of PLVAP in glioma-based immunological responses together with these immunosuppressive processes indicates that PLVAP-targeting precise therapies are within the bounds of probability, and this provides an available premise in regard to coordinating locally aberrant immunity activities, obstructing immunosuppressive invasion, and raising glioma sensitivity towards treatments [57].

Lastly, we conceived PLVAP as a practicable marker and a promising target for glioma-relevant precise diagnosis as well as therapeutic strategies. PLVAP-based diagnostic and treatment strategies have been applied to many diseases. Non-invasive liquid biopsies based on PLVAP have been used for early diagnosis and therapy delamination of hepatocellular carcinoma (HCC) [58]. Fluorescently labeled antibodies against PLVAP were identified as sensitive targeted imaging tracers for melanoma [31]. Anti-PLVAP antibodies have been demonstrated to be effective in regard to treating endotoxin-mediated inflammation [59], pulmonary fibrosis [25], kidney disease [25] and certain retinal diseases [49]. The recombinant monoclonal anti-PLVAP antibody caused tumor vascular thrombosis and necrosis of HCC in a xenograft model [32]. PLVAP-targeted therapy has been demonstrated to inhibit the growth of cholangiocarcinoma [19], lung cancer [28], and pancreatic carcinoma by decreasing tumor angiogenesis [30, 32]. Novel nanocarriers targeting PLVAP provide a beneficial platform for the caveola-dependent delivery of precise targeting biotherapeutics [16, 28]. PLVAP is currently considered a possible therapeutic target for gliomas, glioma-related encephaloedema, and secondary intracranial hypertension [9, 26, 33, 34]. As a promising antiangiogenic target, PLVAP may serve as a specific molecule via which caveola-mediated pathological processes can be regulated in gliomas [26, 33]. It has been demonstrated that PLVAP is remarkably related to contrast enhancement on T1-weighted sequences of GBM, and this correlates with the prognosis and recurrence of patients. Thus, PLVAP may further be used for the diagnosis and treatment optimization of radiologic characteristics of gliomas [35, 60]. PLVAP-targeted neutralizing antibodies, small molecule inhibitors, transcription inhibitors, or blockers identifying interacting proteins with PLVAP may be combined with antibody-enzyme conjugates, conventional nanocarriers, or new biomimetic nanocarriers to achieve precise delivery [32, 37, 40, 61]. For example, a novel recombinant monoclonal anti-PLVAP antibody along with PLVAP-binding antibodies fragments may be used for anti-angiogenic therapy in gliomas [32, 62]. PLVAP-targeted ferritin nanocarriers and lysozyme-dextran nanogel nanocarriers have been demonstrated to provide effective and precise targeting [16, 28], as ferritin nanocarriers can easily pass through the BBB while loading glioma-targeted drugs [63, 64]. Interestingly, in light of these reports and together with our observations, PLVAP-targeting iatreusis is likely to supplement present therapeutic tactics for glioma, whether administered singly or in combination with immunotherapies, and it could also be used in the context of anti-angiogenesis therapeutics. In the meantime, regarding these deficiencies among cellulous statistics based on TCGA and CGGA databases, PLVAP-relevant monocellular investigations of gliomas are currently being conducted. Elucidating the specific mechanism underlying the function of PLVAP in glioma-associated immunosuppression remains a challenge. In the future, PLVAP is expected to be applied for molecule-integrated diagnosis, comprehensive therapeutics, and fluorescence molecule imaging during operations and in the construction of targeted drug carriers for glioma.

## 4 Conclusions

Briefly, our explorations primarily investigated PLVAP expression patterns, biological functions, and clinical value in the context of glioma. Here, we determined the correlations of PLVAP high expression levels with neoplasm-infiltrated immunocytes, immunosuppressive processes, unsatisfactory survival, and pernicious pathology types among gliomas. More importantly, we found that PLVAP is markedly related with immunosuppressive immunocytes infiltration and inflammatory responses in glioma, which greatly extends the understanding about the immune properties of PLVAP. These findings raise the prospects of PLVAP as an encouraging marker and likely target for glioma-based precise diagnosis, therapies, and prognosis evaluations. We anticipate that PLVAP-targeting treatments, either individual or in combination with comprehensive therapies, will become a consequential tactic for combined and individual precise therapies for glioma.

## 5 Materials and methods

### 5.1 Patients and samples

A total of 1024 patients diagnosed with WHO grade II-IV gliomas were included in this retrospective observational study. Among them, 699 glioma patients in the TCGA set were classified as the training set (Supplementary Table 6), while the other 325 glioma patients in the CGGA set were classified as the validation set (Supplementary Table 7). We downloaded the total RNA-seq data, socio-demographic data, pathological grading information, molecular pathology information, and survival time of former training sets from the website http://cancergenome.nih.gov/, while the corresponding data for the latter validation set were obtained from http://www.cgga.org.cn. We included cases that met following inclusion criteria: the TCGA and CGGA cases diagnosed with glioma according to the diagnostic criteria of the fourth edition of the WHO classification of CNS tumors; the cases had intact RNA-Seq level 3 (normalized) data, socio-demographic data, pathological grading information, molecular pathology information, and survival information. We excluded cases based on following exclusion criteria: the cases whose pathological diagnosis does not conform to glioma; the re-enrolled cases of recurrent glioma; the cases lacking any information of RNA-Seq data, socio-demographic data, pathological grading data, molecular pathology data, or survival data. All the information was downloaded, extracted, preprocessed and reviewed by two masked independent investigators. Another two experienced investigators independently checked and analyzed above information. Seventy-eight glioma patients who lacked intact data were removed after primary assessment, and these included 63 patients in the training set and 15 patients in the validation set. Finally, the training set (female 268(42.1%), male 368(57.9%); the median age at initial diagnosis was 47 years (range 14–89 years)) enrolled 223 cases of WHO II(35.1%), 245 cases of WHO III(38.5%) and 168 cases of WHO IV(26.4%). The validation set (female 115(37.1%), male 195(62.9%); the median age at initial diagnosis was 43 years (range 8–81years)) enrolled 105 cases of WHO II(33.9%), 67 cases of WHO III(21.6%), 138 cases of WHO IV(44.5%). We obtained approval (counterpart number: S2020018) from the Ethics Committees of Peking University Third Hospital.

### 5.2 Statistical analysis

Structures predicted using the Alpha Fold machine-learning algorithm were conducted on the website https://alphafold.ebi.ac.uk/. Other statistical analyses together with figure visualization were performed using RStudio software for MacOS, version 1.3.1093 (https://www.rstudio.com/) with several application packages obtained from the website http://www.r-project.org. The difference of PLVAP expression was analyzed and visualized with “devtools” and “ggpubr” packages. Kaplan–Meier survivorship curvilinear analyses together with multivariable Cox analyses were performed using “survival” and “survminer” packages followed by the log-rank test to compare survival differences among the included patients. Kaplan–Meier method was constructed based on the mean of PLVAP expression to stratify patients. Hazard ratio and 95% confidence interval were assessed with the Cox regression model. Akaike information criterion (AIC) and concordance index (CI) were applied to evaluate the superiority and accuracy of the model. ROC curves were performed using “pROC” package to show the value of PLVAP in evaluating TCGA molecular subtypes. Spearman correlation analyses with “psych” package were used for sequencing and for sifting genes that were markedly related to PLVAP. Gene ontology function analysis of gene biological processes and molecular functions together with cellular components was conducted via the website of DAVID Bioinformatical Resource (https://david.ncifcrf.gov/) and visualized through “ggpubr” and “ggplot2” packages. Venn-diagram performed using “ggvenn” package was to show the overlapped genes of the two databases. AmiGO2 version 2.5.17 was utilized for downloading analyzing immunogene subsets to investigate the functions of PLVAP among glioma-associated immunity responses (http://amigo.geneontology.org/amigo). Cluster analysis of RNA expression was performed using “pheatmap” package. Correlograms performed using “corrgram” and “corrplot” packages were used for visualizing the correlation between PLVAP and inflammatory metagenes. Correlation scatter diagrams with “ggplot 2” and “ggpubr” packages were also applied for correlation visualization.

Logarithmic transformations were applied to the transcriptome sequencing data that were analyzed in this study prior to further analysis. The normality of data was tested by Shapiro-Wilk. Bartlett test was used to inspect the homogeneity of variance of all the data. The difference testing for each of the two continuous normally distributed statistical clusters was completed using independent samples Student’s t test. Differences comparisons of two and three or more groups of non-normally distributed variables were analyzed, respectively, using the nonparametric Mann-Whitney U-test and Kruskal-Wallis H test with Dunn post hoc tests (R package “FSA”). The Bonferroni correction was used to control the type I error rate for multiple testing. Spearman method was applied for correlational degree assessments of non-normally distributed variables. Two-tailed P values <0.05 were considered to be statistically significant.

## Declarations

### Author contribution statement

Kaiming Ma: Conceived and designed the experiments; Performed the experiments; Analyzed and interpreted the data; Contributed reagents, materials, analysis tools or data; Wrote the paper.

Xin Chen: Analyzed and interpreted the data; Contributed reagents, materials, analysis tools or data.

Xiaofang Zhao; Suhua Chen: Performed the experiments; Analyzed and interpreted the data.

Jun Yang: Conceived and designed the experiments; Contributed reagents, materials, analysis tools or data; Wrote the paper.

### Funding statement

Prof. Jun Yang was supported by 10.13039/501100001809National Natural Science Foundation of China [82072774 & 81872051], 10.13039/501100007937Peking University Clinical Scientist Program [BMU2019LCKXJ007], Key Clinical Projects of 10.13039/501100009399Peking University Third Hospital [BYSY2018060].

Xin Chen was supported by 10.13039/501100002858China Postdoctoral Science Foundation [2020M670064], 10.13039/501100004826Beijing Natural Science Foundation [7214271].

### Data availability statement

Data will be made available on request.

### Declaration of interests statement

The authors declare no conflict of interest.

### Additional information

Supplementary content related to this article has been published online at https://doi.org/10.1016/j.heliyon.2022.e10298.

## Figures and Tables

**Fig. S2:**