# Supplementary material for: PLVAP is associated with glioma-associated malignant processes and immunosuppressive cell infiltration as a promising marker for prognosis
Source: Heliyon. 2022 Aug 19;8(8):e10298. doi: 10.1016/j.heliyon.2022.e10298 (PMC9404362; doi:10.1016/j.heliyon.2022.e10298)
Supplement: Multimedia component 1 [file mmc1.pdf]

Supplementary Table 1. Genes associated with PLVAP in TCGA and CGGA datasets.
There are 188 related genes from TCGA database (170 positively corelated genes, 18 negatively corelated genes) and 226 related genes from CGGA database (206 positively corelated genes, 20 negatively corelated genes).

TCGA Dataset		CGGA Dataset	
Genes	R	Genes	R
SLC6A1	−0.64	HLF	−0.66
HLF	−0.64	PPM1K	−0.65
CRY2	−0.62	IGIP	−0.65
DZIP3	−0.62	FUT9	−0.64
MADD	−0.62	NAP1L3	−0.63
KIF3A	−0.62	ENHO	−0.63
CPEB3	−0.62	ARHGEF26−AS1	−0.63
NDRG3	−0.62	GABARAPL2	−0.62
NAP1L3	−0.62	ADHFE1	−0.62
TOM1L2	−0.62	CAMK2G	−0.62
TEF	−0.62	KLHDC1	−0.62
PFKM	−0.61	KIAA1107	−0.62
CASKIN1	−0.61	IL17D	−0.61
RIC3	−0.61	FP588	−0.61
SGSM1	−0.61	GLUD1	−0.61
TMEM151B	−0.61	RAB6B	−0.61
TRIM23	−0.61	TSPAN7	−0.61
SPTAN1	−0.61	FBXL17	−0.61
APEX2	0.61	PTPN4	−0.61
FCGRT	0.61	PNMAL2	−0.61
NCKAP1L	0.61	EHD2	0.61
SQRDL	0.61	RNPEP	0.61
TNFRSF12A	0.61	GTSE1	0.61
PIK3AP1	0.61	ANXA2	0.61
CTSS	0.61	ACTN1	0.61
IKBIP	0.61	ADM	0.61
PARVG	0.61	PCDH18	0.61
SAMSN1	0.61	PRKCDBP	0.61
KDELR1	0.61	BCL3	0.61
PTPN7	0.61	CDK2	0.61
PYGL	0.61	ADAM19	0.61
PDPN	0.61	GRWD1	0.61
SERPINB8	0.61	NPM1	0.61
ARPC2	0.61	TRPC6	0.61
EHD4	0.61	OLFML1	0.61
BCL2L12	0.61	POPDC2	0.61
FPR3	0.61	CHSY1	0.61
LAIR1	0.61	RAB8A	0.61
DDOST	0.61	SHKBP1	0.61
LHFPL2	0.61	S100A11	0.61
PDIA5	0.61	MSR1	0.61
ADPRH	0.61	DCBLD2	0.61
COL4A1	0.61	JAG1	0.61
ST8SIA4	0.61	CREB3L2	0.62
SLC11A1	0.61	GPR124	0.62
A2M	0.61	LRRC59	0.62
C1orf38	0.61	HEYL	0.62
CTSB	0.61	MOB3A	0.62
PLEK	0.61	ITGA11	0.62
LY96	0.61	EIF4E2	0.62

TCIRG1	0.61	TUBA1C	0.62
MMP14	0.61	HK3	0.62
RHOH	0.61	TNFRSF12A	0.62
ARID5A	0.61	GRAP	0.62
B4GALT1	0.61	ACTA2	0.62
CCR1	0.61	STAB1	0.62
RELB	0.61	CDH5	0.62
CISH	0.61	TMED9	0.62
KIAA1949	0.61	PPIB	0.62
LTBR	0.61	EVA1B	0.62
C1orf144	0.61	WDR1	0.62
PYCARD	0.61	MYL9	0.62
HM13	0.61	PVRL2	0.62
LYN	0.61	TNFRSF1A	0.62
HLA-DMB	0.61	RARS	0.62
AK2	0.61	ZDHHC18	0.62
MGAT1	0.61	CHPF2	0.62
SLC10A3	0.61	MCM5	0.63
FBP1	0.61	ASF1B	0.63
RAB42	0.61	STK10	0.63
FTL	0.61	AGRN	0.63
BAK1	0.61	ACTB	0.63
FAM70B	0.61	KDELR1	0.63
LOXL2	0.61	AFAP1L1	0.63
GAL3ST4	0.61	EIF4EBP1	0.63
ALOX5AP	0.61	FAM114A1	0.63
STAC3	0.61	RBMS1	0.63
CD4	0.62	GNS	0.63
IL4I1	0.62	ATP8B1	0.63
STAB1	0.62	GBE1	0.63
GPR65	0.62	CDC20	0.63
EMR2	0.62	TNKS1BP1	0.63
SLC1A5	0.62	IFRD2	0.63
PTPN6	0.62	TMEM45A	0.63
PPP1CA	0.62	ITGA4	0.63
CTSZ	0.62	MYO1G	0.63
TRIM38	0.62	FHOD1	0.63
CYTH4	0.62	PDIA3	0.63
LEPRE1	0.62	TXNDC5	0.63
TMSL3	0.62	HSPA5	0.63
FCGBP	0.62	SPRY1	0.63
ITGA5	0.62	IQGAP3	0.63
ARL11	0.62	IFI30	0.63
HK3	0.62	PPP1CA	0.63
RBM47	0.62	ETV6	0.63
C1QA	0.62	TOR4A	0.64
TYROBP	0.63	CCDC86	0.64
IFNGR2	0.63	LUM	0.64
SHKBP1	0.63	RIPK1	0.64
LCP1	0.63	DNAJB11	0.64
PLOD1	0.63	CNN2	0.64
CD40	0.63	TACC3	0.64
STXBP2	0.63	PDLIM1	0.64

CD53	0.63	MXRA5	0.64
CYBA	0.63	COL6A1	0.64
ABI3	0.63	DPP9	0.64
PLAU	0.63	FLNA	0.64
C5AR1	0.63	PLK1	0.64
ITGB2	0.63	NID1	0.65
RAB32	0.63	REEP4	0.65
GPR160	0.63	GJA4	0.65
CCRL2	0.63	TRAM2	0.65
VSIG4	0.63	FSTL1	0.65
CASP4	0.63	FLT4	0.65
HAVCR2	0.63	SERPINE1	0.65
SASH3	0.63	LXN	0.65
ADPGK	0.63	ATF5	0.65
PHC2	0.63	ARHGDIB	0.65
PLIN2	0.64	CSDA	0.65
TYMP	0.64	PRSS23	0.65
GPSM3	0.64	TBXA2R	0.65
CAPZA1	0.64	TGFBI	0.65
SERPINA1	0.64	ACTN4	0.66
HCK	0.64	KIF20A	0.66
RPS6KA1	0.64	TIMP1	0.66
C2	0.64	IL10RB	0.66
CD300C	0.64	SHCBP1	0.66
ARPC5	0.64	BAK1	0.66
IL10RB	0.64	CKAP4	0.66
FCGR3A	0.64	FAM20C	0.66
REEP4	0.64	ARPC5	0.66
FCGR2A	0.64	GLA	0.66
CLIC1	0.64	THBD	0.66
CDCP1	0.64	ITGB1	0.66
DOK3	0.65	DDOST	0.66
PLAUR	0.65	COL15A1	0.66
GRN	0.65	PLXDC1	0.66
C17orf87	0.65	PLOD3	0.66
SLC7A7	0.65	C19orf10	0.66
VAMP8	0.65	RPN1	0.66
LRRC25	0.65	COL5A2	0.67
SPI1	0.65	ADAMTS7	0.67
FHOD1	0.65	RCN3	0.67
TGFB1	0.65	IGFBP2	0.67
P2RX4	0.65	ST14	0.67
GLT25D1	0.65	PLEKHG2	0.67
FPR1	0.65	SRPX2	0.67
CMTM7	0.65	HMOX1	0.67
SPP1	0.66	GPX8	0.67
KCNQ1	0.66	TUBB6	0.67
ELF4	0.66	SEC61A1	0.67
NFAM1	0.66	SEC24D	0.67
GMFG	0.66	TPM4	0.67
MSR1	0.66	PCOLCE	0.67
FCGR1A	0.66	LAMC3	0.68
C1QB	0.66	LMAN2	0.68

LCP2	0.66
C1QC	0.66
SLC39A1	0.66
TMEM173	0.66
LAPTM5	0.67
NAGA	0.67
FERMT3	0.67
CD14	0.67
ST14	0.67
RAC2	0.67
CD68	0.67
C9orf167	0.67
UCP2	0.67
ADAP2	0.68
CD276	0.68
IFI30	0.68
RUNX1	0.68
FCER1G	0.68
S100A11	0.68
RGS19	0.68
CTSC	0.69
SH2B3	0.69
SIGLEC9	0.69
PFN1	0.69
OLFML3	0.7
ARPC1B	0.7
HMOX1	0.7
ARHGDIB	0.71
CD300A	0.71
KCNE3	0.71
VASP	0.73
SLC16A3	0.77

CLIC1	0.68
EMILIN1	0.68
SNAI2	0.68
PDIA4	0.68
SUSD2	0.68
OLFML2B	0.68
TK1	0.68
IGFBP4	0.68
PRDX4	0.68
TMEM255B	0.69
NOTCH3	0.69
MMP14	0.69
MANF	0.69
ACE	0.69
COL6A2	0.69
PCDH12	0.69
TMEM8A	0.69
ITGB3	0.69
FRMD8	0.69
ANPEP	0.69
GUSB	0.69
NDUFA4L2	0.7
IKBIP	0.7
DLL4	0.7
KDELR2	0.7
CD276	0.7
ZDHHC5	0.7
HTRA3	0.7
GRN	0.7
PLXND1	0.7
SH2B3	0.7
VKORC1	0.7
CALR	0.7
HLX	0.71
GPR4	0.71
SYDE1	0.71
C1QTNF6	0.72
MYH9	0.72
EHD4	0.72
PECAM1	0.72
SERPINH1	0.72
VASP	0.73
GLT25D1	0.73
EXOC3L1	0.74
ADAM12	0.74
LAMB1	0.74
PMM2	0.74
PFN1	0.74
MMP11	0.74
PLOD1	0.74
MCAM	0.75
LAMC1	0.75
COL1A2	0.75

KCNE3	0.75
ENPEP	0.75
BGN	0.75
SEMA3F	0.75
P4HB	0.76
LOXL2	0.76
FN1	0.77
COL1A1	0.77
CD248	0.78
NID2	0.78
ITGA5	0.78
COL3A1	0.78
ITGA1	0.78
CD93	0.79
HSPG2	0.81
COL4A2	0.82
COL4A1	0.82
