# Supplementary material for: PLVAP is associated with glioma-associated malignant processes and immunosuppressive cell infiltration as a promising marker for prognosis
Source: Heliyon. 2022 Aug 19;8(8):e10298. doi: 10.1016/j.heliyon.2022.e10298 (PMC9404362; doi:10.1016/j.heliyon.2022.e10298)
Supplement: Multimedia component 2 [file mmc2.pdf]

Supplementary Table 2. Overlapped 35 PLVAP-related genes of TCGA and CGGA datasets.

Overlapped related genes
HLF
NAP1L3
TNFRSF12A
IKBIP
KDELR1
EHD4
DDOST
COL4A1
MMP14
BAK1
LOXL2
STAB1
PPP1CA
ITGA5
HK3
SHKBP1
PLOD1
ARPC5
IL10RB
REEP4
CLIC1
GRN
FHOD1
GLT25D1
MSR1
ST14
CD276
IFI30
S100A11
SH2B3
PFN1
HMOX1
ARHGDIB
KCNE3
VASP
