# Supplementary material for: PLVAP is associated with glioma-associated malignant processes and immunosuppressive cell infiltration as a promising marker for prognosis
Source: Heliyon. 2022 Aug 19;8(8):e10298. doi: 10.1016/j.heliyon.2022.e10298 (PMC9404362; doi:10.1016/j.heliyon.2022.e10298)
Supplement: Multimedia component 3 [file mmc3.pdf]

Supplementary Table 3. Immune-related genes significantly related to PLVAP in TCGA and CGGA datasets.

There are 100 immune-related genes in the TCGA database and 60 immune-related genes in the CGGA database which was significantly corrected with PLVAP.

TCGA database Genes	CGGA database Genes
KIF3A	FUT9
TRIM23	CAMK2G
SPTAN1	IL17D
FCGRT	ANXA2
NCKAP1L	ACTN1
CTSS	BCL3
SAMSN1	S100A11
PYGL	JAG1
PDPN	HK3
ARPC2	PPIB
FPR3	WDR1
LAIR1	MYL9
DDOST	STK10
SLC11A1	ACTB
A2M	GNS
CTSB	ITGA4
PLEK	MYO1G
LY96	PDIA3
TCIRG1	TXNDC5
MMP14	IFI30
RHOH	ETV6
ARID5A	RIPK1
B4GALT1	CNN2
CCR1	FLNA
RELB	FLT4
LTBR	SERPINE1
PYCARD	IL10RB
LYN	BAK1
HLA-DMB	CKAP4
FTL	ARPC5
BAK1	GLA
CD4	THBD
IL4I1	ITGB1
GPR65	DDOST
PTPN6	IGFBP2
CTSZ	HMOX1
TRIM38	SEC24D
ITGA5	EMILIN1
HK3	PRDX4
RBM47	MMP14
C1QA	ACE
TYROBP	ITGB3
IFNGR2	ANPEP
LCP1	GUSB
CD40	DLL4
STXBP2	CD276
CD53	GRN
CYBA	SH2B3

PLAU
C5AR1
ITGB2
RAB32
CCRL2
VSIG4
CASP4
HAVCR2
SASH3
GPSM3
CAPZA1
SERPINA1
HCK
C2
CD300C
ARPC5
IL10RB
FCGR3A
FCGR2A
DOK3
PLAUR
GRN
SLC7A7
VAMP8
SPI1
TGFB1
P2RX4
FPR1
CMTM7
ELF4
NFAM1
GMFG
FCGR1A
C1QB
LCP2
C1QC
LAPTM5
CD14
RAC2
CD68
CD276
IFI30
RUNX1
FCER1G
S100A11
CTSC
SH2B3
SIGLEC9
ARPC1B
HMOX1
CD300A
SLC16A3

CALR
HLX
MYH9
PECAM1
COL1A2
FN1
COL1A1
CD248
ITGA5
COL3A1
ITGA1
CD93
