# Supplementary material for: PLVAP is associated with glioma-associated malignant processes and immunosuppressive cell infiltration as a promising marker for prognosis
Source: Heliyon. 2022 Aug 19;8(8):e10298. doi: 10.1016/j.heliyon.2022.e10298 (PMC9404362; doi:10.1016/j.heliyon.2022.e10298)
Supplement: Multimedia component 5 [file mmc5.pdf]

Supplementary Table 5. Detailed information about the immune cell specific marker genes. There are some specific marker genes of CD4+ T cells, regulatory T cells (Tregs), CD8+ T cells, tumor-associated macrophages (TAMs), myeloid-derived suppressor cells (MDSCs) and neutrophils (NEUT).

Cells	Markers
Macrophages	CD14
Macrophages	HLADRA
Macrophages	CD312
Macrophages	CD115
Macrophages	CD163
Macrophages	CD204
Macrophages	CD301
Macrophages	CD206
Neutrophils	CD11b
Neutrophils	CD16
Neutrophils	CD66b
Neutrophils	ELANE
MDSCs	CD14
MDSCs	CD16
MDSCs	CD33
MDSCs	ARG1
CD8T	CD3E
CD8T	CD8A
NK	CD16
NK	CD56
Tregs	CD3E
Tregs	CD4
Tregs	CD25
Tregs	FOXP3
CD4T	CD3E
CD4T	CD4
